# Expression and role of microRNA-212/nuclear factor I-A in depressive mice

**DOI:** 10.1080/21655979.2021.2009964

**Published:** 2021-12-10

**Authors:** Liang Si, Yanyan Wang, Min Liu, Lifeng Yang, Li Zhang

**Affiliations:** a Department of Psychiatry, Wuhan Mental Health Center, Wuhan, China; b Department of Psychiatry, Affiliated Wuhan Mental Health Center, Tongji Medical College of Huazhong University of Science & Technology, Wuhan, China

**Keywords:** miR-212, depression, chronic unpredictable mild stress, hippocampus, NFIA

## Abstract

Depression is characterized by persistent depressed mood and cognitive dysfunction, severely impacting human health. In the present study, we aimed to explore the role and mechanism of microRNA (miR)-212 in depression *in vivo*. Chronic unpredictable mild stress (CUMS) mice were established, and depression-like behaviors were confirmed using the forced swimming test (FST), sucrose preference test (SPT), and the tail suspension test (TST). Next, the expression of miR-212 and its potential target, i.e., nuclear factor I-A (NFIA), was verified using quantitative reverse transcription (qRT)-PCR analysis and Western blotting in CUMS mice. The effects of miR-212 and NFIA on depression-like behaviors, inflammatory response, and neuronal apoptosis were examined using FST, TST, SPT, enzyme-linked immunosorbent assay (ELISA) assay, and flow cytometry analysis. Finally, the relationship between miR-212 and NFIA was examined using a dual-luciferase reporter assay. Based on our findings, miR-212 was significantly upregulated, while NFIA was downregulated in CUMS mice. miR-212 overexpression could suppress the CUMS-induced weight loss, immobility time in FST and TST, and increased hippocampal neuronal apoptosis and pro-inflammatory cytokines levels. In addition, NFIA upregulation could partially reverse the effects of miR-212 mimic in CUMS mice. Accordingly, miR-212 could ameliorate CUMS-induced depression-like behavior in mice by targeting NFIA, indicating its protective role in depression.

## Introduction

Depression is one of the most common psychiatric disorders, primarily characterized by the patient’s depressed mood, loss of sense of enjoyment or interest in the outside world, self-perception of incompetence or inadequacy, depressed psychological needs, and disconnection from society [1]. These manifestations might persist for prolonged periods or frequently recur in individuals experiencing depression, thereby severely affecting the individual’s ability to work, study, or perform day-to-day activities [2]. Mild depression can be managed without medication. In contrast, individuals with moderate or severe depression may require medication and more specialized treatment [3]. In 2017, the World Health Organization reported that worldwide depression was estimated to exceed 300 million people, representing approximately 4.4% of the world’s population [4]. According to the latest report, the number of people suffering from depression in China accounts for 4% of the total population. Accordingly, depression is currently considered a major disease endangering the health and quality of life of the general population [5]. However, the types, manifestations, causes, and complementary treatments for depression remain extremely complex and diverse, and the complementary treatments cannot be generalized. Hence, it was imperative to identify new approaches for treating depression.

In recent years, there have been more and more studies on the diagnosis and treatment bio-markers of depression [6,7]. Over the last few years, microRNAs (miRNAs) have become research hotspots in various diseases. Moreover, a large number of miRNAs were found to be specifically expressed in the brain or enriched in the central nervous system. For instance, miR-21 was shown to be poorly expressed in hypoxia-ischemic brain damage rats, indicating a protective role in reducing cerebral infarct volume and brain damage [8]. Another study elucidated that miR-233 could promote neuronal repair while inhibiting neuroinflammation in neurodegenerative diseases [9]. Moreover, miRNAs reportedly participate in synaptic development, dendritic protein synthesis, axon guidance and neuronal formation by regulating the translation process of mRNAs [10–13]. In addition, miRNAs have been associated with stress-induced mood disorders [14,15]. Accumulating evidence indicates that miRNAs play important roles in regulating depression, including miR-323a, miR-207 and miR-34a [16–18]. However, studies examining miRNAs for treating depression remain in the preliminary stage, and the mainstream strategy aimed to employ miRNAs as biomarkers for depression diagnosis or antidepressant drugs. In the present study, we investigated the effects of miR-212 in an animal model of depression-like behavior.

Studies have shown that mice in a chronically stressed state display symptoms similar to those exhibited by patients with depression, such as anxious behavior, reduced socialization, and lack of pleasure (anhedonia) [19,20]. Thus, over the years, chronic unpredictable mild stress (CUMS) models have been validated for psychological and behavioral studies to establish chronic stress-related animal models of human depression and depressive symptoms, which have become valuable tools for evaluating depression as well as co-morbid disorders by reproducing clinical symptoms of human depression, including anhedonia and learned helplessness [21–23]. Moreover, CUMS models are now widely used to perform pharmacological studies, assessing the pathogenesis of depression, as well as antidepressant drugs [21,24].

Studies have demonstrated the role of miR-212 in neurological-related diseases [25–28]. For example, Weigelt et al. [25] have reported the down-regulation 0 f miR-212 in patients with postpartum psychosis. Guan et al. [26] have revealed thet miR-212-3p improved functional recovery of spinal cord injury rats by targeting PTEN. miR-212-5p attenuates ferroptotic neuronal death after traumatic brain injury [27]. miR-212 promotes the recovery function in mice with ischemic stroke [28]. Moreover, study has shown that after treatment with antidepressants, the level of miR-212 in the serum of patients increases significantly [29]. Another study showed that the expression of miR-212 was significantly increased in CUMS-induced mice [30]. But its specific role and mechanism of miR-212 in depression is still unclear. Bioinformatics analysis revealed a direct binding site between miR-212 and NFIA. NFIA plays key critical role in central nervous system development and brain function [31], and it has been reported to play important roles in the regulation of cell apoptosis and inflammatory response [32].

We hypothesized that miR-212 might play a role in depression via regulating NFIA. Hence, in the present study, we established a CUMS mouse model as previously described and performed an in-depth assessment of the miR-212-mediated mechanism in depression.

## Materials and methods

### Establishment of CUMS mice and depression-like behaviors [33]

C57BL/6 mice (male, 8–10 weeks; weight, 18–22 g) were purchased from the Nanjing University Animal Research Center and received a standard diet of pellet chow. All experimental procedures were in accordance with the regulations of the Animal Use and Management Committee of the Wuhan Mental Health Center.

In addition, animals had free access to water and were maintained on a 12–12 h light/dark cycle. The model was established over a 6-week period, during which mice were administered continuous stress and received different mild stresses each day to prevent anticipation and adaptation to the stress mode. The stressors included: (1) food deprivation for 24 h, (2) water deprivation for 24 h, (3) overnight lighting, (4) cage without wood chips for 24 h, (5) wetting wood chips with water for 24 h, (6) forced swimming in 8°C for 5 min, (7) tail clamping (1 cm from tail tip), (8) physical restraint for 6 h, and (9) 45° cage tilt with vertical axis for 3 h. After treatment, the mice were anesthetized with chloral hydrate and then decapitated. Brain tissues and peripheral samples were quickly harvested on ice and washed with phosphate-buffered saline (PBS), with brains dissected on ice dishes. Approximately 3 mm^3^ from the left side of the cerebral cortex was preserved for flow cytometric detection of apoptosis, and the remaining tissue was placed into Eppendorf tubes® and frozen at −80°C for subsequent detection of relevant genes and proteins.

To examine the role of miR-212 in depression, CUMS mice were divided into the following groups: control group (unstressed regular feeding), CUMS group (CUMS treatment), CUMS + mimic control treatment group (CUMS mice were intraperitoneally injected with mimic control), CUMS + miR-212 mimic treatment group (CUMS mice were intraperitoneally injected with miR-212 mimic), CUMS + miR-212 mimic + control-plasmid treatment group (CUMS mice were intraperitoneally injected with miR-212 mimic + control-plasmid), CUMS + miR-212 mimic + NFIA-plasmid treatment group (CUMS mice were intraperitoneally injected with miR-212 mimic + NFIA-plasmid), and positive control group (CUMS mice were intraperitoneally injected with fluoxetine [FLU] 20 mg/kg/d).

### Sucrose preference test (SPT)

Sucrose preference is an indicator of the animals’ preference for sugar water and the pursuit of ‘pleasure’, indicating that the animal was in a more rational state physically and mentally. A double bottle feeding method was used (one bottle provided 1% sucrose water [100 mL], and the other provided normal drinking water [100 mL]). To prevent mouse dependence on sucrose water, bottle positions were exchanged every 12 h. After 72 h, the consumption of sucrose and drinking water was measured, and sucrose preference was calculated as follows: Sucrose preference (%) = sucrose water consumption/(sucrose water consumption + pure water consumption) × 100% [33].

### Forced swimming test (FST)

The test animals were placed in a 24 cm high, 12 cm diameter Plexiglas cylinder tank, with a water depth of 20 cm and a water temperature of 25 ± 2°C. A suitably placed camera recorded the animal swimming for 6 min, and the cumulative immobility time was measured for the next 4 min. Animals were considered immobile when they stopped struggling and floated in the water for 2 s. Animals were acclimatized in the behavioral laboratory for at least 1 h before each experiment, ensuring that the time was consistent for each experiment. At the end of the experiment, body temperature was restored using a heat lamp, and the animals were then returned to their respective cages [34].

### Tail suspension test (TST)

The mice were suspended upside down by tape at a height of 30 cm, approximately 1 cm from the tail tip, to ensure that there was no possibility of climbing and grasping from front to back. Mice were considered immobile when completely motionless. Using a camera, the experiment was recorded for 6 min, and the cumulative immobility time was measured over the next 4 min. Animals were acclimatized in the behavioral laboratory for at least 1 h before each experiment, ensuring that time was consistent for each experimen [35].

### Quantitative reverse transcription-PCR (qRT-PCR) analysis

TRIzol reagent was used for total RNA extraction from peripheral blood samples and hippocampal tissues. Total RNA was measured using the OneDrop spectrophotometer (Beijing, China). RNA was reverse transcribed to cDNA, and PCR amplification was performed using qPCR SYBE Green Master Mix on an ABI Step One Plus system. Briefly, amplification cycles were as follows: initial denaturation at 95°C for 5 min, 40 cycles of denaturation at 95°C for 15 sec and annealing/elongation at 60°C for 30 sec, final extension at 60°C for 60 s. The primer sequences (Sangon Biotech, Shanghai, China) were as follows: miR-212 forward, 5ʹ-GCCTCCTGACTCCAGGTCC-3ʹ, and reverse, 5ʹ-GCGCAAAGTGACTGGATGAA-3ʹ; U6 forward, 5ʹ-CTCGCTTCGGCAGCACATATACT-3ʹ, and reverse, 5ʹ-ACGCTTCACGAATTTGCGTGTC-3ʹ; NFIA forward, 5ʹ-ACCAGCTCAAAAAACCTGTGGA-3ʹ, and reverse, 5ʹ-TGTTGTGAAACGAAACACCCC-3ʹ; GAPDH forward, 5ʹ-CATCAAGAAGGTGGTGAAGCAG-3ʹ, and reverse, 5ʹ-CGTCAAAGGTGGAGGAGTGG-3ʹ. Target gene expression was quantified using the 2^−ΔΔCt^ method [36]

### ELISA assay [37]

Briefly, 100 mg of hippocampal tissues were placed in fetal bovine serum (PBS) and homogenized thoroughly. Then, the supernatant was collected by centrifugation at 5000 × g at 4°C for 5 min. Cytokine levels (interleukin [IL]-1β, IL-6, and tumor necrosis factor [TNF]-α) were measured using instructions provided by the corresponding ELISA kits.

### Flow cytometry analysis [38]

In brief, 3 mm^3^ fresh cerebral cortex was digested with 0.25% trypsin without Ethylene Diamine Tetraacetic Acid (EDTA) for about 30 min to obtain a single-cell suspension. The cell density was adjusted to approximately 5 × 10^5^, and the corresponding reagents were added according to the instructions provided by the Annexin V-FITC/PI Apoptosis Detection Kit. The reaction was performed at room temperature for 10 ~ 15 min, and the cells were detected on the flow cytometer within 1 h.

### Western blotting [39]

Total proteins were extracted from neuronal cells using radioimmunoprecipitation assay (RIPA) lysis and measured using the bicinchoninic acid (BCA) method. In brief, 12% sodium dodecyl sulfate-polyacrylamide gel electrophoresis (SDS-PAGE) was utilized to separate proteins, then transferred onto polyvinylidene fluoride (PVDF) membranes. After blocking in 5% skimmed milk for 50 min, membranes were incubated with primary antibodies (rabbit anti-cleaved-Caspase3, 1:1000, ab2302; rabbit anti-NFIA, 1:500, ab179928; rabbit anti-GAPDH, 1:2500, ab9485; Abcam, Shanghai, China) at 4°C overnight. After 24 h, the membranes were incubated with secondary antibody (IgG H&L DyLight®488, 1:1000, ab96899) and enhanced chemiluminescent reagent. Finally, Chemiluminescence Gel Imaging System and ImageJ software (National Institutes of Health, Bethesda, MD) were employed to analyze bands relative to the internal control.

### Dual-luciferase reporter assay [40]

Briefly, 293 T cells were purchased from American Type Culture Collection (ATCC; USA) and cultured in Dulbecco’s Modified Eagle Medium with 10% PBS and 1% penicillin-streptomycin under 5% CO_2_ and at 37°C. The targeted fragment of NFIA in miR-212 was cloned from the genomic DNA by bioinformatics and then inserted into the luciferase reporter gene plasmid (psiCHECK-2) to construct the NFIA-wild type (wt) plasmid. In addition, the corresponding mutant control-plasmid NFIA-mut was prepared and purified by GenePharma (Shanghai, China). In 293 T cells, the reporter plasmids were co-transfected with miR-212 mimic or mimic control for 48 h. Finally, the luciferase activity was measured and evaluated relative to *Renilla* activity.

## Statistical analysis

Data values are expressed as mean ± standard error (SD) and analyzed using SPSS 22.0 (IBM Corp., Armonk, NY, USA). Student’s t-test or one-way ANOVA was applied for analyzing the data. A P value <0.05 was established as statistically significant.

## Results

### miR-212 expression was markedly induced in CUMS mice

**Figure 1.**
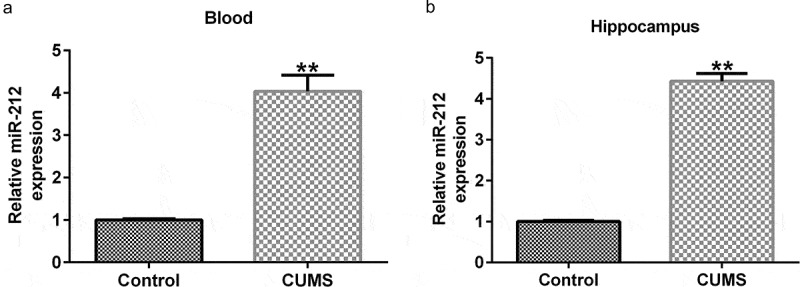
**Expression of miR-212 in CUMS mice**. The expression level of miR-212 in peripheral blood samples (a) and hippocampus tissues (b) in CUMS mice was determined using quantitative reverse transcription (qRT)-PCR. **p < 0.01 *vs*. Control group. CUMS, Chronic unpredictable mild stress

To confirm the underlying mechanism of miR-212 in depression, we first established CUMS-induced depression mice. As shown in Figure 1(a–b), miR-212 was markedly increased in peripheral samples and hippocampal tissues extracted from CUMS mice when compared with the unstressed control group.

### miR-212 alleviated weight loss and depression-related behaviors in CUMS-induced mice

**Figure 2.**
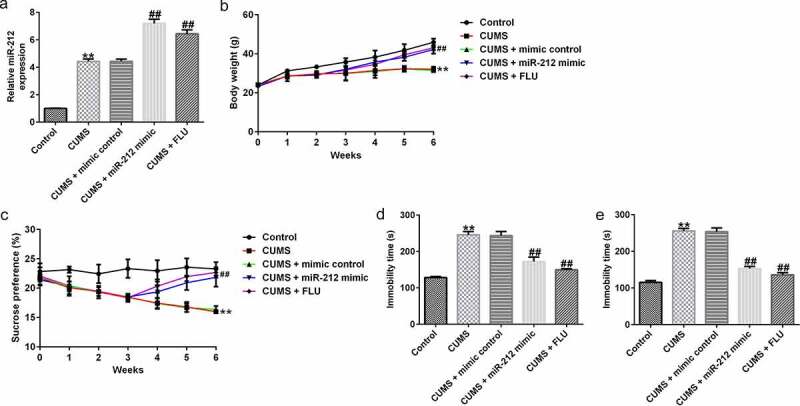
**miR-212 mitigates body weight loss and depression-like behaviors in CUMS-induced mice**. (a) Expression of miR-212 in hippocampal tissues of mice after treatment with miR-212 mimic was measured using quantitative reverse transcription (qRT)-PCR. (b) Body weight of mice in different groups. (c) miR-212 mimic improves the sucrose preference in CUMS model. (d) miR-212 mimic shortens the immobility in forced swimming test in CUMS mice. (e) miR-212 mimic reduces the immobility in tail suspension test in CUMS mice. **p < 0.01 *vs*. Control group; ##p < 0.01 *vs*. CUMS+mimic control group. CUMS, Chronic unpredictable mild stress

Next, we examined the role of miR-212 in depression-like behaviors in CUMS mice. As shown in Figure 2(a), compared with the control group, CUMS-induced model group presented increased miR-212 expression; furthermore, following transfection with the miR-212 mimic, miR-212 levels were increased when compared with the mimic control treatment group. Furthermore, miR-212 ameliorated CUMS-mediated weight loss (Figure 2(b)). In terms of depression-live behaviors, as shown in Figure 2(c–e), the miR-212 mimic improved the sucrose preference while shortening the immobility time in FST and TST when compared with the control group. In addition, the effects of miR-212 on CUMS-induced body weight loss and depression-like behaviors were similar to those mediated by FLU treatment (Figure 2(b–e)).

### Protective role of miR-212 on neuronal cell apoptosis in CUMS mice

**Figure 3.**
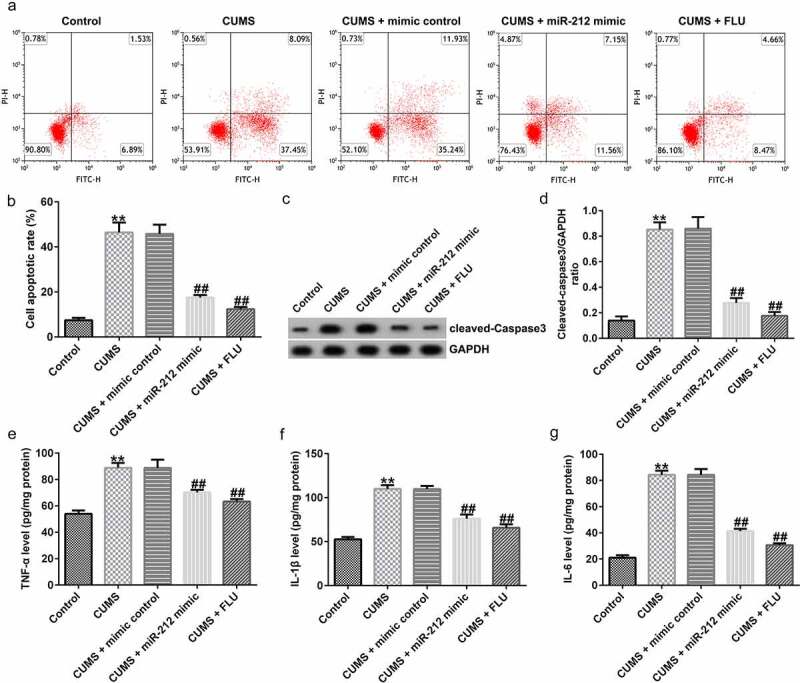
**miR-212 ameliorates CUMS-induced neuronal cell apoptosis and inflammation response in the hippocampus**. (a-b) Cell apoptosis rate measured by flow cytometry analysis. (c-d) Cleaved-Caspase3 expression levels and cleaved-Caspase3/GAPDH ratio in CUMS mice after treatment with miR-212 mimic or FLU were detected by Western blot assay. Expression levels of TNF-α (e), IL-1β (f), and IL-6 (g) in CUMS hippocampus were evaluated using ELISA. **p < 0.01 *vs*. Control group; ##p < 0.01 *vs*. CUMS+mimic control group. CUMS, Chronic unpredictable mild stress; FLU, fluoxetine; IL-1β, interleukin-1β; Il-6, interleukin-6; TNF-α, tumor necrosis factor-α

To explore the role of miR-212 in neuronal cell apoptosis in CUMS mice, after extraction of neuronal cells from the hippocampus of CUMS mice, the effects of miR-212 on neuronal cell apoptosis were examined using flow cytometry and Western blotting. As shown in Figure 3(a–b), neuronal apoptosis was increased in CUMS-induced mice when compared with the control group; however, miR-212 overexpression and FLU treatment could partially counteract the increased CUM-induced apoptosis. Meanwhile, elevated levels of protein cleaved-Caspase3 and cleaved-Caspase3/GAPDH ratio in CUMS-induced mice were antagonized by the miR-212 mimic or FLU treatment (Figure 3(c–d)). Collectively, miR-212 afforded protection against neuronal cell apoptosis in the depression mouse model.

### miR-212 mitigated the CUMS-induced inflammatory response in the hippocampus

To explore the role of miR-212 in CUMS-induced inflammatory response in the hippocampus, the inflammatory response was quantified by measuring hippocampal expression levels of inflammation-related cytokines in different groups. As shown in Figure 3(e–g), compared with the unstressed control group, CUMS treatment increased expression levels of TNF-α, IL-1β, and IL-6; however, administering the miR-212 mimic and FLU decreased TNF-α, IL-1β, and IL-6 levels when compared with the CUMS model group.

### NFIA was negatively regulated by miR-212 in CUMS mice

**Figure 4.**
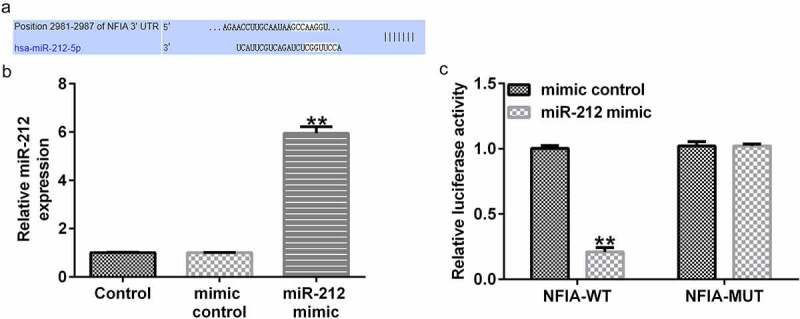
**NFIA is a downstream target of miR-212**. (a) Potential binding sites between NFIA and miR-212 were predicted by the TargetScan online tool. (b) miR-212 level in 293 T cells transfected with mimic control or miR-212 mimic was measured using quantitative reverse transcription (qRT)-PCR. (c) Dual-luciferase reporter assay confirmed the targeted relationship between miR-212 and NFIA. **p < 0.01 *vs*. mimic control group

TargetScan software predicted the targeted sites between miR-212 and NFIA (as shown in Figure 4(a)). Furthermore, the dual-luciferase reporter assay was used to confirm potential binding sites. Compared with the mimic control group, miR-212 was significantly enhanced in miR-212 mimic transfected 293 T cells (Figure 4(b)). As shown in Figure 4(c), the dual-luciferase reporter assay illustrated that co-transfection with miR-212 mimic and NFIA-WT plasmid significantly reduced luciferase activity when compared with mimic control and NFIA-WT plasmid co-transfection group. However, no significant differences were detected in the luciferase activity of cells co-transfected with miR-212 mimic and NFIA-MUT and those co-transfected with the mimic control and NFIA-MUT.

**Figure 5.**
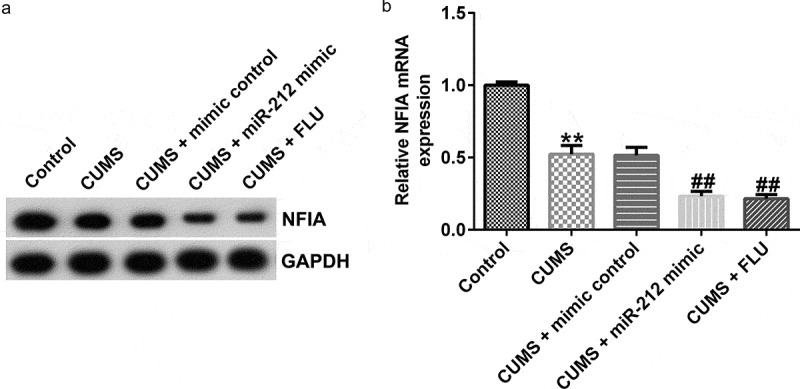
**NFIA is negatively regulated by miR-212 in CUMS-induced mice**. (a) NFIA protein expressions in CUMS mice with or without miR-212 mimic treatment were determined by Western blotting. (b) NFIA mRNA expression levels in CUMS-induced mice with or without miR-212 mimic treatment were detected by quantitative reverse transcription (qRT)-PCR analysis. **p < 0.01 *vs*. Control group; ##p < 0.01 *vs*. CUMS+mimic control group. CUMS, Chronic unpredictable mild stress

Moreover, following CUMS treatment, NFIA protein and mRNA expression were significantly decreased in hippocampus tissues extracted from the CUMS model group when compared with the control group; however, administration of miR-212 mimic and FLU decreased NFIA levels when compared with CUMS model group (Figure 5(a–b)).

### miR-212 improved depression-like behaviors and neuronal cell apoptosis by targeting NFIA in CUMS mice

**Figure 6.**
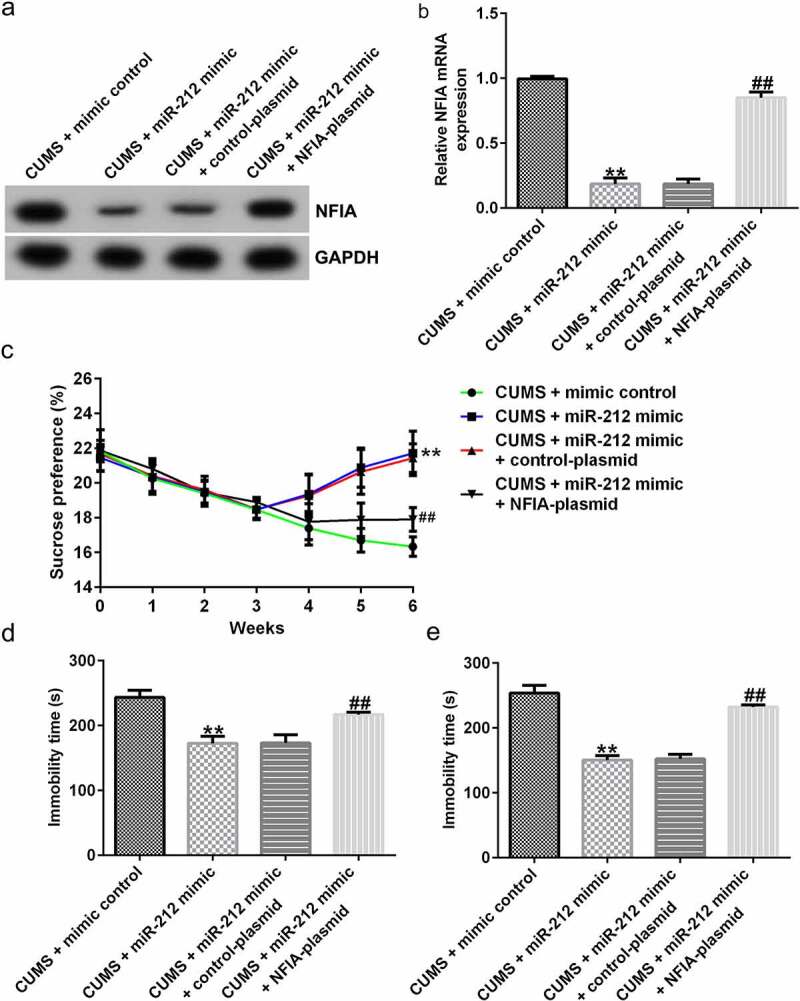
**miR-212 ameliorates depression-like behaviors in CUMS-induced mice by targeting NFIA**. (a) Protein expression levels of NFIA in different CUMS groups. (b) NFIA mRNA expressions levels in different CUMS groups. (c) NFIA overexpression reduces the sucrose preference in CUMS mice when compared with those treated with miR-212 mimic. (d-e) NFIA worsens the immobility time in forced swimming and tail suspension tests in CUMS mice when compared with the miR-212 mimic + control-plasmid treatment group. **p < 0.01 *vs*. CUMS + mimic control group; ##p < 0.01 *vs*. CUMS + miR-212 mimic + control-plasmid group. CUMS, Chronic unpredictable mild stress

**Figure 7.**
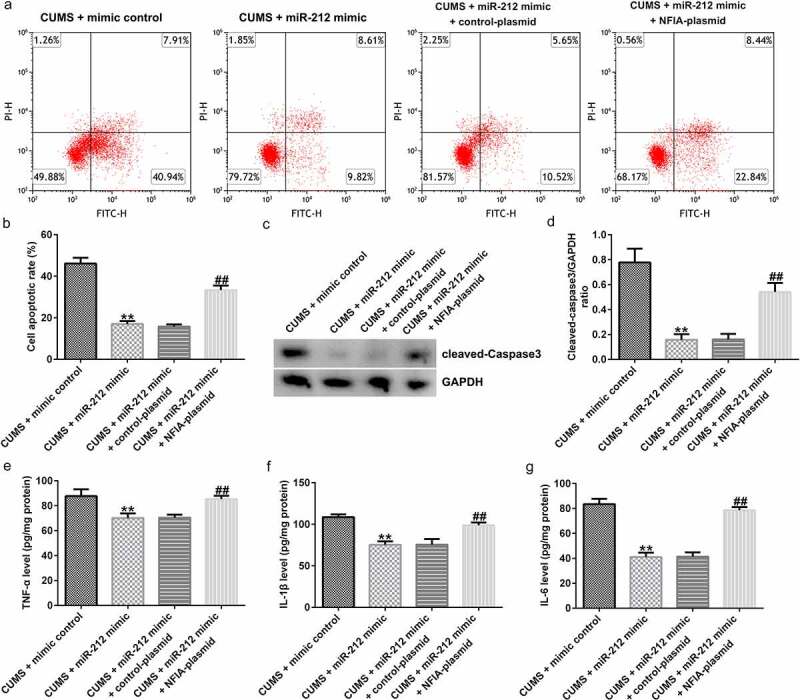
**miR-212 suppresses neuronal cell apoptosis and inflammation response in CUMS hippocampus by targeting NFIA**. (a-b) Cell apoptosis rate in different groups. (c-d) Cleaved-Caspase3 and cleaved-Caspase3/GAPDH3 ratio were determined by Western blotting. (e-g) Concentrations of TNF-α, IL-1β, and IL-6 in CUMS hippocampus were evaluated by ELISA. **p < 0.01 *vs*. CUMS + mimic control group; ##p < 0.01 *vs*. CUMS + miR-212 mimic + control-plasmid group. CUMS, Chronic unpredictable mild stress; IL-1β, interleukin-1β; Il-6, interleukin-6; TNF-α, tumor necrosis factor-α

Gain-of-function experiments further demonstrated the regulatory mechanism of the miR-212/NFIA axis in depression. After establishing the CUMS model, mice were intraperitoneally administered miR-212 mimic, control-plasmid, and NFIA-plasmid. As shown in Figure 6(a–b), both protein and mRNA expression levels of NFIA were significantly increased in the CUMS + miR-212-mimic + NFIA-plasmid group when compared with the CUMS + miR-212-mimic + control-plasmid group. In CUMS-induced mice, the SPT, FST and TST assay results revealed that the miR-212 mimic-mediated improvements in sucrose preference, immobility time in FST and TST could be counteracted by NFIA upregulation (Figure 6(c–e)). Meanwhile, the decreased apoptosis, cleaved-Caspase3 level, and cleaved-Caspase3/GAPDH ratio induced by miR-212 mimic could be reversed by co-transfection with NFIA-plasmid (Figure 7(a-d)). In addition, NFIA overexpression partially suppressed the inhibitory effects of miR-212 mimic on TNF-α, IL-1β, and IL-6 in CUMS-induced mice (Figure 7(e–g)).

## Discussion

Depression is characterized as a disorder of higher neurological functions in the brain. Based on accumulated evidence, organic changes in the central nervous system in patients with depression, and the hippocampus, as part of the limbic system, are responsible for mood regulation and cognitive management functions [41,42]. Numerous clinical and animal experimental studies have reported that the stress response greatly affects the hippocampus [43,44]. In patients with depression, magnetic resonance imaging has revealed that the hippocampus showed volume atrophy, and the degree of alteration correlated with the disease duration and treatment course [45,46]. Moreover, recent reports have suggested that inflammation and neuroapoptosis are the main pathophysiological mechanisms of depression [47,48]. Psychological and social stress could increase the levels of inflammatory cytokines in the body. Notably, increased levels of pro-inflammatory cytokines such as IL-1β, IL-6 and TNF-α were markedly decreased in patients with depression receiving antidepressant treatment [49,50]. Furthermore, recent studies have confirmed the stress-mediated activation of inflammation [51–53]. In the present study, after CUMS treatment, we utilized SPT, FST, and TST assays to verify that the CUMS mice were successfully established. After constructing the CUMS mouse model, we observed that the rate of neuronal apoptosis was significantly increased, along with enhanced expression of IL-1β, IL-6 and TNF-α; these findings were consistent with those of previous studies [54,55].

miRNAs are a class of non-coding, single-stranded small molecule RNAs, approximately 22 nucleotides length, widely involved in regulating cell proliferation, differentiation, and apoptosis by regulating mRNAs [56]. It has been reported that miRNAs have specific distribution and development in the brain and other neural tissues [54,57]. Numerous studies have shown that miRNAs are involved in various neuropsychiatric disorders, including schizophrenia and anxiety [58,59]. Furthermore, it has been reported that miRNAs play important roles in the nervous system and regulate neuronal development, differentiation, and synaptic plasticity [60–62]. Fan et al. have documented that five miRNAs were upregulated in peripheral blood mononuclear cells derived from patients with depression, thus serving as sensitive and specific biomarkers for depressive disorders [63]. In addition, studies in animal depression models found that specific miRNAs in hippocampal brain regions could regulate neuroinflammatory responses, thus improving depressive symptoms [64,65]. A previous study by Lin et al. has reported that the miR-212 level was markedly increased after 4 weeks of antidepressant treatment [29]. Electroconvulsive therapy (ECT) is considered an effective antidepressant treatment. Ryan et al. have reported that the miR-212 level was significantly improved in the rat dentate gyrus following acute and chronic ECT treatment [66]. However, the specific role of miR-212 in regulating depression development remains unclear. Consistent with a previous study [30], the present study found that miR-212 was substantially enhanced in the CUMS model group when compared with the unstressed control group. Furthermore, we revealed for the first time that miR-212 mimic could ameliorate the CUMS-induced body weight loss, depression-like symptoms, and inflammation response. Also, miR-212 mimic significantly reduced neuronal cell apoptosis, which was consistent with previous research [67]. These data suggested a protective role for miR-212 in depression development. Moreover, the targeted relationship between miR-212 and NFIA was verified using the dual-luciferase reporter assay. Finally, functional experiments revealed that the protective effects of miR-212 in the CUMS depression model could be partially counteracted by NFIA overexpression, thus confirming the regulatory role of the miR-212/NFIA axis in depression development.

However, there were some limitations of current study. First, we did not analyze the expression of miR-212/NFIA in patients with depression, and the correlation between miR-212/NFIA expression and clinicopathological parameters in patients with depression. In addition, we did not study the effect of NFIA alone on mice with depression. We will perform these issues in the future.

## Conclusion

miR-212 might play a protective role in depression by regulating apoptosis and inflammatory responses in mouse hippocampal neurons by targeting NFIA. Thus, miR-212 may be a promising diagnostic and therapeutic target for depression clinical treatment.

## Data Availability

All data sets used and/or generated during the current study are available from the corresponding author on reasonable request.

## Research highlights

1) miR-212 expression was markedly induced in CUMS mice;

2) NFIA was negatively regulated by miR-212 in CUMS mice;

3) miR-212 improved depression-like behaviors and neuronal cell apoptosis by targeting NFIA in CUMS mice;
